# Surgery of the Upper Extremities

**Published:** 1889-04

**Authors:** Edward M. Moore

**Affiliations:** 74 South Fitzhugh Street; Rochester, N. Y.


### *SURGERY OF THE UPPER EXTREMITIES.*

### PART III.

### FRACTURES OF THE SCAPULA.

BY EDWARD M. MOORE, M. D., LL.D., Rochester, N. Y.

The rarity of these fractures, has rendered it difficult to indicate any one plan of treatment with the assurance of its being better than any other. We may find the bone broken in a variety of conditions, some of which are simple of diagnosis, while others are almost impossible of accurate definition. When the body is broken below the spine, the fracture is easy of recognition, as it is also when the acromion is the part thus injured. But fractures of the neck of the scapula are very difficult to diagnosticate. Fortunately, they are very rare. I am not sure that I have ever seen a case. Fractures of the coracoid are also very infrequent, and, although the point stands out well, much doubt has ever hung over the question of diagnosis.

The first case that I ever saw of fracture of scapula was one where the line was transverse through the body. The patient did not come into my hands until the process of repair was well advanced in the fourth week. The gentleman who was so unfortunate as to suffer from this fracture, was thrown from his berth by the lurch of the ship on his voyage to New York from Europe. He, on his arrival in the city, at once applied to Prof. J. M. Carnochan, who fitted a Dessault clavicle bandage to him. But the relief obtained was slight. The sharp lancinating pain, so characteristic of this fracture, was very great. No motion of the body was made that did not increase the sufferings. No change in the treatment was made, and it was many months before relief was obtained.

The case that has interested me more than any other, I must report at second hand, for I did not see the man who was hurt, though he lived but a few miles from my residence. He was a laborer on the railroad, who, while walking by the side of the track, received a blow from the projecting beam that constitutes the frame of the locomotive. The engine passed him and struck him upon the scapula, throwing him into the ditch. The pain was very severe. He was carried into a hut near by, and sent for medical aid. This was furnished from the neighboring village, but no attempt at treatment was made, for the doctor did not have "the right kind of splints." The next morning a young physician, Dr. Rogers, went out from Rochester to examine the case. He found the man absolutely unwilling to move, or be moved, on account of the severe pain. After not a little trouble, he was raised to the sitting posture, when the diagnosis of fracture of the scapula transversely below the spine, was made out. I use Dr. Rogers' language: "Now came the puzzle. I had not the least idea what should be done. So I thought I would try your clavicle bandage." He further stated that the transformation of the man's behavior was marvelous. He at once began to move, and found that the pain had nearly disappeared. After getting on his feet and walking a little, he started off on foot and went to his own hut, half a mile away. He made a good recovery. I have seen a similar case since this was told me, which came into my hands about a week after the accident. There was a greal deal of pain, and while the clavicle bandage gave great relief, it was not so marked as in the case narrated above. The inflammatory process was already established.

The superiority of the "figure eight from the elbow" over other forms of the clavicle bandage will, I think, become apparent, not only from these cases, but from the theory of its action. By pressing the humerus backwards, the scapula is slid around on the thorax, and brought into close contact with it, thus affording a splint of the natural kind. Besides this primary necessity, a more complete fixation of the scapula is obtained by the binding of the arm, which prevents its motion. The two ends of the bandage press upon the scapula as they pass to the opposite shoulder, thus making the outside substitute for a splint. But there is still more to be said with reference to the aid derived from the position of the humerus. When the elbow is carried backward, the teres major, which is inserted in the edge of the bicipital groove of the humerus, and arises from the lower portion of the body of the scapula, is rendered lax by this position. This prevents the tension of the most powerful muscle liable to separate the fragments.

Of fractures of the neck of the scapula, I can say but little. I have the testimony of one physician only, who became satisfied that the case he had was a fracture of the neck. The treatment was by the use of the "elbow figure eight," and the result remarkably good. I saw the patient at the termination of convalescence, about eight weeks after the accident. The result was quite perfect. That the opposition of the broken fragments would be likely to be assured, would be inferred from the fact that the shoulder is raised, but cannot carry the neck too high from the intervention of the acromion. The pressure against the body of the scapula to move it towards the spine, must keep the fragments in close contact, while the general action of the bandage seems quiet.

That the apparatus under discussion would be the proper one in cases of fracture of the acromion, must be admitted, because the forces of displacement are essentially those that control fractures of the clavicle.

It will thus be seen that I regard the "elbow figure eight" as preëminently fitted for the treatment of all fractures of the scapula, except those of the coracoid. Where this process is broken off, the treatment is pretty obvious. The humerus should be carried forward, and the forearm well flexed upon the arm, to secure relaxation of the coraco-brachialis and the biceps flexor-cubiti.

I have purposely left out of consideration all questions of diagnosis. These can be settled by reference to the proper sources of information.

74 South Fitzhugh Street.

